# Reducing TRPC1 Expression through Liposome-Mediated siRNA Delivery Markedly Attenuates Hypoxia-Induced Pulmonary Arterial Hypertension in a Murine Model

**DOI:** 10.1155/2014/316214

**Published:** 2014-12-18

**Authors:** Cheuk-Kwan Sun, Yen-Yi Zhen, Hung-I Lu, Pei-Hsun Sung, Li-Teh Chang, Tzu-Hsien Tsai, Jiunn-Jye Sheu, Yung-Lung Chen, Sarah Chua, Hsueh-Wen Chang, Yi-Ling Chen, Fan-Yen Lee, Hon-Kan Yip

**Affiliations:** ^1^Department of Emergency Medicine, E-Da Hospital, I-Shou University College of Medicine, Kaohsiung 82445, Taiwan; ^2^Division of Cardiology, Department of Internal Medicine, Kaohsiung Chang Gung Memorial Hospital and Chang Gung University College of Medicine, Kaohsiung 83301, Taiwan; ^3^Division of Thoracic and Cardiovascular Surgery, Department of Surgery, Kaohsiung Chang Gung Memorial Hospital and Chang Gung University College of Medicine, Kaohsiung 83301, Taiwan; ^4^Basic Science, Nursing Department, Meiho Institute of Technology, Pingtung 91202, Taiwan; ^5^Department of Biological Sciences, National Sun Yat-sen University, Kaohsiung 80424, Taiwan; ^6^Center for Translational Research in Biomedical Sciences, Kaohsiung Chang Gung Memorial Hospital and Chang Gung University College of Medicine, Kaohsiung 83301, Taiwan; ^7^Institute of Shock Wave Medicine and Tissue Engineering, Kaohsiung Chang Gung Memorial Hospital and Chang Gung University College of Medicine, Kaohsiung 83301, Taiwan

## Abstract

We tested the hypothesis that Lipofectamine siRNA delivery to deplete transient receptor potential cation channel (TRPC) 1 protein expression can suppress hypoxia-induced pulmonary arterial hypertension (PAH) in mice. Adult male C57BL/6 mice were equally divided into group 1 (normal controls), group 2 (hypoxia), and group 3 (hypoxia + siRNA TRPC1). By day 28, right ventricular systolic pressure (RVSP), number of muscularized arteries, right ventricle (RV), and lung weights were increased in group 2 than in group 1 and reduced in group 3 compared with group 2. Pulmonary crowded score showed similar pattern, whereas number of alveolar sacs exhibited an opposite pattern compared to that of RVSP in all groups. Protein expressions of TRPCs, HIF-1*α*, Ku-70, apoptosis, and fibrosis and pulmonary mRNA expressions of inflammatory markers were similar pattern, whereas protein expressions of antifibrosis and VEGF were opposite to the pattern of RVSP. Cellular markers of pulmonary DNA damage, repair, and smooth muscle proliferation exhibited a pattern similar to that of RVSP. The mRNA expressions of proapoptotic and hypertrophy biomarkers displayed a similar pattern, whereas sarcomere length showed an opposite pattern compared to that of RVSP in all groups. Lipofectamine siRNA delivery effectively reduced TRPC1 expression, thereby attenuating PAH-associated RV and pulmonary arteriolar remodeling.

## 1. Introduction

In daily clinical practice, patients with pulmonary arterial hypertension have been currently categorized into five classes according to different etiologies of the disease [1]: pulmonary arterial hypertension (group 1); pulmonary hypertension due to left heart disease (group 2); pulmonary hypertension due to chronic lung disease and/or hypoxia (group 3); chronic thromboembolic pulmonary hypertension (group 4); and pulmonary hypertension due to unclear multifactorial mechanisms (group 5).

Besides, there are also a variety of conditions associated with pulmonary hypertension, including collagen vascular disease, chronic obstructive lung disease, consequence of adult respiratory distress syndrome, hepatopulmonary syndrome (portal hypertension), recurrent pulmonary embolism, congenital (intracardiac shunt) and valvular heart disease, and primary pulmonary arterial hypertension (PAH) [2]. PAH is a progressive and ultimately fatal disease mainly because of the obliteration and/or degeneration of distal precapillary pulmonary arterioles [3–5]. Without treatment, the life expectancy of patients suffering from the disease is extremely short (i.e., an average of 2.8 years from diagnosis) [3–7].

Traditionally, the treatment strategies for PAH include long-term oxygen therapy or inhalational nitric oxide [8], vasodilators [9], calcium channel blockers [10], intravenous prostacyclin [11, 12], and endothelin antagonists [13, 14], as well as phosphodiesterase inhibitor such as sildenafil [15, 16]. However, all of these therapeutic options remain unsatisfactory due to high cost, limited effectiveness, or serious side effects. To develop a satisfactory therapeutic strategy, therefore, is a formidable challenge to clinicians. To achieve this goal, a deeper understanding of the mechanistic basis of PAH is mandatory.

The transient receptor potential cation channel (TRPC) gene, which was first cloned in Drosophila [17], encodes a light-activated ion channel in photoreceptors. It is widely distributed in various cells and tissues [18–20] and can be activated multimodally through diverse mechanisms. Its mammalian homologs constitute a superfamily of cation channels which comprises 6 subtypes (TRPC1 to TRPC6) [18, 21]. More importantly, previous studies have demonstrated that TRPC1 and TRPC6 play a particularly crucial role in regulating smooth muscle cell contraction and proliferation in the murine model of hypoxia-induced PAH [21].

Genetic medicine has been proposed to be of tremendous therapeutic potential in treating a variety of diseases ranging from cancer [22, 23] to hemophilia [24]. A key hurdle to its clinical application is the lack of safe and effective delivery systems [25, 26]. Development of a safe and effective delivery system, therefore, is of utmost importance in preclinical translational research and also its subsequent clinical application.

The virus-based gene transduction system has a significantly higher efficiency in gene delivery; however, the unwelcome immune-activation limits its development in clinical application [27]. The liposome-mediated nucleic acid delivery system is one of the popular non-virus-based methods to transfer plasmid DNA/siRNA oligos into cells for overexpressing of interfering expression of specific genes [28, 29]. Recently the liposome-mediated gene delivery system has been widely used in experimental animal model to determine* in vivo* gene function and to evaluate the therapeutic efficacy of gene therapy [30, 31]. Therefore, it is believed that this mediator will become an important tool for gene or drug delivery via liquid-spread inhalation in the near future. By using the Lipofectamine Transfection Reagent, this study tests the hypothesis that siRNA may effectively deplete TRPC1 protein expression, thereby suppressing hypoxia-induced PAH in a murine model based on the results of our previous study that highlighted the important roles of TRPC1 and TRPC6 in hypoxia-induced PAH [21].

## 2. Materials and Methods

### 2.1. Ethics

All animal experimental procedures were approved by the Institute of Animal Care and Use Committee at Chang Gung Memorial Hospital-Kaohsiung Medical Center (Affidavit of Approval of Animal Use Protocol number 2008121108) and performed in accordance with the Guide for the Care and Use of Laboratory Animals (NIH publication number 85-23, National Academy Press, Washington, DC, USA, revised 1996).

### 2.2. Animal Model of Chronic Hypoxia-Induced PAH and Grouping

On day 0, 30 pathogen-free, adult male C57BL/6 mice (12-week-old), weighing 22–25 g (Charles River Technology, BioLASCO Co., Ltd., Taiwan), were utilized in the current study. Briefly, the mice were kept in a hypoxia chamber that consisted of (1) a plastic chamber with a built-in electric fan for mixing nitrogen and oxygen, a cage of mice with free access to water and animal chow, barium hydroxide lime for absorbing carbon dioxide, and charcoal powder for eliminating ammonia; (2) an oximeter showing the oxygen content inside the chamber which was consistently kept at 11 ± 0.2%; (3) gas tanks (i.e., nitrogen and air) connected to the chamber for maintaining the hypoxic environment inside the chamber; (4) transparent glass window for monitoring animal activities.

**Figure 1:**
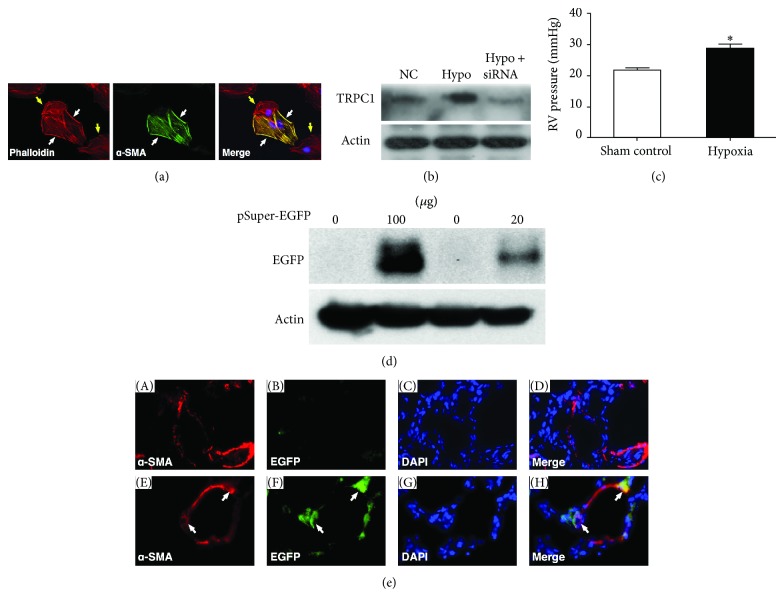
*In vitro* and* in vivo* pilot studies validating the murine model of pulmonary arterial hypertension (PAH). (a) Immunofluorescent studies demonstrating successful primary cell culture using the aortic smooth muscle cells (SMCs) from murine aorta, showing SMCs (white arrows) and non-SMCs. (b) Western blot analysis (*n* = 3) of primary SMC culture showing markedly suppressed transient receptor potential cation channel (TRPC) 1 protein expression in SMCs after siRNA-knockdown (20 nM), suggesting successful knockdown of TRPC1 expression with Lipofectamine delivered siRNA. (c) Significantly elevated right ventricular systolic pressure (RVSP) in animals by 14 days after hypoxia treatment compared to that in normal controls, indicating successful establishment of the murine model of pulmonary arterial hypertension. ∗ versus control, *P* = 0.0054 (*n* = 4 for each group). (d) Western blot and (e) IF analyses (*n* = 3) of lung tissues demonstrating successful pulmonary delivery of Lipofectamine delivered enhanced green fluorescence protein plasmid (pEGFP) through inhalation. (d) Western blot analysis showing enhanced EGFP expression as pSuper-EGFP concentration increased. (e) IF demonstrating (B) control plasmid without EGFP expression in lung tissue and (H) the merged image, indicating successful Lipofectamine transfection of EGFP plasmid into the pulmonary arteriolar (PA) SMCs (white arrows). Blue fluorescence indicates DAPI-stained nuclei. *α*-SMA: alpha-smooth muscle actin staining.

To confirm whether hypoxia-induced PAH in mice was successfully created in this setting, four mice were sacrificed by day 14 of hypoxia. The results showed that the right ventricular systolic pressure (RVSP), an index of pulmonary artery systolic pressure (PASP), was remarkably increased in mice exposed to hypoxia compared to that in normal controls (Figure 1(c)) (*P* = 0.0054). Therefore, the murine model of chronic hypoxia-induced PAH was validated in our pilot study.

Accordingly, the animals were categorized into normal controls (group 1, *n* = 10), hypoxia (for 28 days) + Lipofectamine delivered negative control siRNA (Dharmacon) (group 2, *n* = 10), and hypoxia + Lipofectamine delivered siRNA for depleting TRPC1 (i.e., hypoxia + siRNA) (Dharmacon) (group 3, *n* = 10). Animals in group 1 received an intratracheal spray of 100 *μ*L normal saline, while those in groups 2 and 3 received 100 *μ*L of Lipofectamine delivery of siRNA on day 14 of hypoxia.

### 2.3. Assessment of Knockdown Efficiency of siRNA-TRPC1 by Lipofectamine Delivery in Mouse Aortic Smooth Muscle Cells (SMCs)

To confirm the efficacy of Lipofectamine transfection of siRNA-TRPC1 and the impact of siRNA on TRPC1 expression in aortic SMCs (i.e., primary cell culture from mouse aorta-derived SMCs), Lipofectamine siRNA-TRPC1 was transfected into aortic SMCs for 72 hours, followed by collecting the cells for Western blot analysis (Figure 1(b)). TRPC1 and TRPC3 through TRPC6 channels have been found to be widely expressed in human vessels of all calibers, from the largest conduit vessels to medium size coronary arteries, cerebral arteries, smaller sized resistance arteries, and vasa vasorum [32]. This is the reason why we utilized the mouse aorta-derived SMCs for Lipofectamine delivery of siRNA-TRPC1 in the present experimental setting.

### 2.4. Assessment of* In Vivo* Efficiency of Lipofectamine Transfection in Delivering Enhanced Green Fluorescence Protein (pEGFP) Plasmid DNA into Murine Lung Parenchyma via Intratracheal Administration

To elucidate the transfection efficiency of Lipofectamine-based gene delivery, an enhanced green fluorescent protein (EGFP) containing plasmid DNA (i.e., pSuper-EGFP) was introduced into the lungs through an intratracheal spray. Briefly, 50 *μ*g plasmid DNA and Lipofectamine 5 *μ*L in 100 *μ*L opti-MEM medium were well mixed prior to intratracheal administration. Three C57BL/6 mice aged 12 weeks were anaesthetized. A small spatula was used to open the lower jaw of the mouse and blunted forceps were used to help displace the tongue for maximal oropharyngeal exposure. The 100 *μ*L DNA transfection mixture previously loaded into a MicroSprayer aerosoliser (IA-1C; Penn-Century, Philadelphia, PA, USA) was then carefully sprayed endotracheally after insertion of the tip of the aerosoliser through the larynx. The mice were allowed to recover and then sacrificed 3 days after treatment with the lungs collected for Western blot analysis (Figure 1(d)) and immunofluorescent staining (Figure 1(e)).

Similarly, for TRPC1 gene knockdown, 60 pmole mouse TRPC1-knockdown oligo [Catalog number J-043863 purchased from Dharmacon RNA Technologies (CO, USA)] and 5 *μ*L Lipofectamine were mixed in 100 *μ*L opti-MEM medium. The knockdown oligo mixture was then intratracheally sprayed to the lungs of the mice.

### 2.5. Hemodynamic Assessment and Specimen Preparation

The protocol and procedure of hemodynamic measurement have previously been described [33–36]. On day 28 of hypoxia, the mice were anesthetized by inhalation of 2.0% isoflurane. After being shaved on the chest, each animal was endotracheally intubated with mechanical ventilatory support in room air using a small animal ventilator (model D-79232, Hugo Sachs Elektronik-Harvard Apparatus GmbH, March, Germany) at a rate of 120 per minute and a tidal volume of 250 *μ*L. The heart was exposed by left thoracotomy. A sterile 25-gauge, soft-plastic coated needle was inserted into the right ventricle and left ventricle of each mouse to measure the RVSP and left ventricular systolic blood pressure, respectively, when heart rate was >400 beats/min. The pressure signals were first transmitted to a pressure transducer (UFI, model 1050, CA, USA) and then exported to a bridge amplifier (ML866 PowerLab 4/30 Data Acquisition Systems; ADInstruments Pty Ltd., Castle Hill, NSW, Australia) where the signals were amplified and digitized. The data were recorded and later analyzed with the LabChart software (ADInstruments). After hemodynamic measurements, the mice were euthanized with the hearts and lungs harvested. For each animal, the whole heart weight, right ventricular weight, lung weight, and body weight were recorded from which the ratio of right ventricle-to-whole heart weight was calculated. The preparation of lung specimens for morphometric analyses was based on our recent reports [33, 34]. Briefly, the left lung was inflated at a constant airway pressure (10–15 mmHg) and fixed with OCT (Tissue-Tek) for immunohistochemical staining. The right lung was then cut into pieces that were either fixed in 4% paraformaldehyde PBS solution before being embedded in paraffin blocks for hematoxylin and eosin (H & E) staining or stored at −80°C for protein and mRNA analyses.

### 2.6. Preparation and Culture of Aortic Smooth Muscle Cells (SMCs)

Twelve-week-old male C57BL/6 mice were anesthetized and sacrificed. The descending aorta in each animal was then excised and immersed into sterile PBS buffer immediately. After removal of the connective tissue, the aorta was cut longitudinally. The endothelial cells were first scrapped with forceps. The pieces of aorta were then placed onto a 60 mm Petri dish and kept in close contact with the Petri dish. These tissues were then cultured in 1 mL of DMEM containing 20% FBS + penicillin (100 unit/mL) + streptomycin (100 *μ*g/mL) in a humidified atmosphere of 5% CO_2_ and 95% air at 37°C. By day 4, outgrowth of aortic smooth cells was observed to be released from the aorta and attached to the bottom of the Petri dish. The SMCs were then cultured in DMEM supplemented with 10% FBS penicillin (100 unit/mL) + streptomycin (100 *μ*g/mL) before being trypsinized and seeded (10^5^ cells) in a 35 mm Petri dish. Finally, the 10^6^ cells were transferred to a 100 mm Petri dish to form a subconfluent monolayer to be observed.

### 2.7. Western Blot Analysis of Lung Tissue

The protocol and procedure of Western blot analysis have previously been described [33–36]. Equal amounts (50 *μ*g) of protein extracts were loaded and separated by SDS-PAGE using acrylamide gradients. After electrophoresis, the separated proteins were transferred electrophoretically to a polyvinylidene difluoride (PVDF) membrane (Amersham Biosciences). Nonspecific sites were blocked by incubation of the membrane in blocking buffer [5% nonfat dry milk in T-TBS (TBS containing 0.05% Tween 20)] overnight. The membranes were incubated with the indicated primary antibodies [TRPC1 (1 : 1500, Abcam), TRPC4 (1 : 600, Abcam), TRPC6 (1 : 1500, Abcam), Bax (1 : 1000, Abcam), caspase-3 (1 : 1000, Cell Signaling), poly(ADP-ribose) polymerase (PARP) (1 : 1000, Cell Signaling), protein expressions of Bcl-2 (1 : 200, Abcam), phosphorylated (p)-Smad3 (1 : 1000, Cell Signaling), p-Smad1/5 (1 : 1000, Cell Signaling), bone morphogenetic protein- (BMP-) 2 (1 : 5000, Abcam), transforming growth factor (TGF)-*β* (1 : 500, Abcam), hypoxia-induced factor- (HIF-) 1*α* (1 : 750, Abcam), vascular endothelial growth factor (VEGF) (1 : 1000, Abcam), Ku-70 (1 : 1000, Abcam), and actin (1 : 10000, Chemicon)] for 1 hour at room temperature. Horseradish peroxidase-conjugated anti-rabbit immunoglobulin IgG (1 : 2000, Cell Signaling) was used as a secondary antibody for one hour at room temperature. The washing procedure was repeated eight times within one hour, and immunoreactive bands were visualized by enhanced chemiluminescence (ECL; Amersham Biosciences) and exposed to Biomax L film (Kodak). For purposes of quantification, ECL signals were digitized using Labwork software (UVP).

### 2.8. Quantification of Alveolar Sacs, Crowded Score, and Arteriolar Muscularization in Lung Parenchyma

Left lung specimens from all animals were fixed in 10% buffered formalin before being embedded in paraffin and sectioned at 5 *μ*m for light microscopic analysis. Hematoxylin and eosin (H & E) staining was performed to determine the number of alveolar sacs according to our recent study [34] in a blind fashion. Three lung sections from each mouse were analyzed and three randomly selected high-power fields (HPFs) (100x) were examined in each section. The number of alveolar sacs was recorded for each animal. The mean number per HPF for each animal was then determined by summation of all numbers divided by 9.

The percentage of crowded area (defined as septal thickness associated with partial or complete collapse of alveoli) in lung parenchyma was determined using H & E staining in a blind fashion and scored as follows: 0 = no detectable crowded area; 1 = <15% of crowded area; 2 = 15–25% of crowded area; 3 = 25–50% of crowded area; 4 = 50–75% of crowded area; 5 = >75%–100% of crowded area/per high-power field (100x). The procedure and protocol were based on our recent report [33].

The detailed procedure and protocol for determining pulmonary arteriolar muscularization (i.e., an index of vascular remodeling) was described in detail in our recent report [34] with minimal modification. Briefly, three measurements were taken for the thickness of pulmonary arterioles. Muscularization of the arterial medial layer in lung parenchyma was defined as a mean thickness of vessel wall greater than 40% of the lumen diameter in a vessel of diameter > 30 *μ*m. Measurement of arteriolar diameter and wall thickness was achieved using the Image-J software (NIH, Maryland, USA).

### 2.9. Immunofluorescent (IF) Studies

The procedures and protocols for IF examination were based on our recent study [37]. Briefly, IF staining was performed for the examinations of phosphorylated variants of histone H2AX (*γ*-H2AX), Ki-67, TRPC1, HIF-1, Ku-70, and troponin-I using respective primary antibodies and irrelevant antibodies were used as controls. Three sections of lung specimens were analyzed in each mouse. For quantification, three randomly selected HPFs (×200 for IF study) were analyzed in each section. The mean number per HPF for each animal was then determined by summation of all numbers divided by 9. An HF-based scoring system was adopted for semiquantitative analyses of these biomarkers in lung as a percentage of positive cells in a blinded fashion (score of positively stained cell for *γ*-H2AX, Ki-67, TRPC1, HIF-1, and Ku-70: 0 = no stain %; 1 = <15%; 2 = 15~25%; 3 = 25~50%; 4 = 50~75%; 5 = >75%–100%/per HPF in PA or in lung parenchyma).

### 2.10. Real-Time Quantitative PCR Analysis for mRNA Expressions in Lung Tissue and Right Ventricle

Real-time polymerase chain reaction (PCR) was conducted using LighCycler TaqMan Master (Roche) in a single capillary tube according to the manufacturer's guidelines for individual component concentrations. Forward and reverse primers (Table 1) were each designed in different exons of the target gene sequence, eliminating the possibility for amplifying genomic DNA. A positive result was determined by identifying the threshold cycle value at which reporter dye emission appeared above background. If fluorescence signal was not detected within 55 cycles, the sample was considered negative.

### 2.11. Statistical Analysis

Quantitative data are expressed as means ± SD. Statistical analysis was adequately performed by ANOVA followed by Bonferroni multiple comparison post hoc test. Statistical analysis was performed using SAS statistical software for Windows version 8.2 (SAS institute, Cary, NC). A probability value <0.05 was considered statistically significant.

## 3. Results

### 3.1. Pilot Studies* In Vitro* and* In Vivo*

The right ventricular blood pressure (RVSP), an indirect indicator of pulmonary arterial systolic pressure (PASP) at day 14, was significantly higher in the hypoxic group than in normal controls (Figure 1(c)). This finding indicates that the murine hypoxia-induced PAH model was successfully created. Western blot analysis of primary aortic SMC culture (Figure 1(a)) showed that the protein expression of TRPC1 was markedly attenuated after siRNA-TRPC1 (20 nM) treatment (Figure 1(b)) suggesting that this method is feasible in knocking down the expressions of TRPC1 in aortic SMCs.

Immunofluorescent microscopic findings showed that EGFP was expressed in the alpha-smooth muscle actin (*α*-SMA) positively stained cells (Figure 1(e)) [i.e., the pulmonary arteriolar smooth muscle cells (PASMCs)]. In addition, Western blot also demonstrated that EGFP was expressed in the pSuper-EGFP-transfected lungs with a dosage-dependent manner (Figure 1(d)).

### 3.2. The Baseline Characteristics of the Three Groups on Day 28 after Hypoxia-Induced PAH

The final body weight did not differ among the three groups. However, the hemoglobulin and red blood cell count were significantly higher in PAH animals (group 2) and those treated with siRNA-TRPC1 (group 3) than in sham controls (group 1), but the two parameters were similar between groups 2 and 3 (Table 2). Moreover, the left ventricular systolic blood pressure, which is an indirect indicator of systolic arterial blood pressure, was similar among the three groups. On the other hand, the right ventricular systolic blood pressure (an indicator of PASP) was significantly higher in the untreated PAH group (group 2) than in the normal controls (groups 1) and the siRNA-treated group (group 3) and significantly higher in group 3 than in group 1 (Table 2).

Gross anatomical findings showed no significant difference in total heart weight among the three groups. However, the RV weight and total lung weight were significantly higher in group 2 than in groups 1 and 3, but no difference was noted between the latter two groups (Table 2). Additionally, the RV-to-total heart weight ratio and the total lung weight were significantly higher in group 2 than those in groups 1 and 3, but the two parameters were similar between groups 1 and 3.

### 3.3. Histopathological Changes in Lung Parenchyma on Day 28 after Hypoxia-Induced PAH

**Figure 2:**
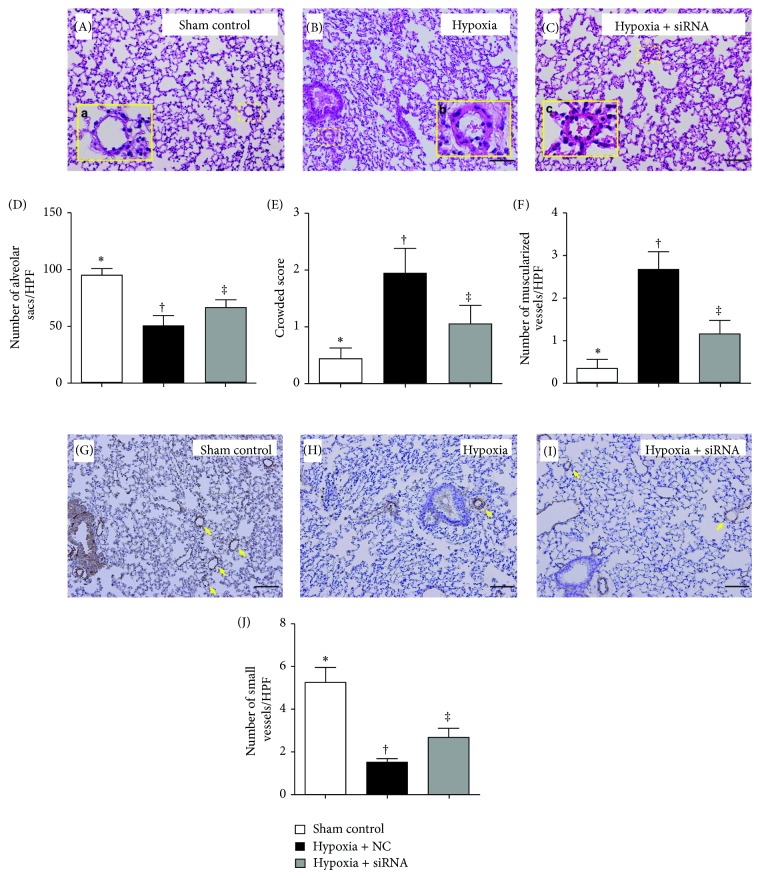
Histopathological and immunohistochemical (IHC) changes in lung parenchyma by day 28 after hypoxia-induced pulmonary arterial hypertension (PAH) (*n* = 10). ((A) to (C)) Hematoxylin and eosin (H & E) staining (100x) showing (A) normal pulmonary arterioles in sham controls, (B) hypertrophic muscularized medial layer of pulmonary arterioles (PA) in hypoxia-treated mice (i.e., dotted-squares magnified as solid-line squares), and (C) PA in hypoxia-treated animals with siRNA treatment. The scale bars in right lower corners represent 100 *μ*m. (D) Number of alveolar sacs among the three groups. ∗ versus other groups with different symbols (∗, †, ‡), *P* < 0.0001. (E) Crowded score of pulmonary tissue among the three groups. ∗ versus other groups with different symbols (∗, †, ‡), *P* < 0.001. (F) Number of muscularized vessels per high-power field (HPF). ∗ versus other groups with different symbols (∗, †, ‡), *P* < 0.001. ((G) to (I)) IHC staining (i.e., *α*-SMA staining) (100x) illustrating the number of small pulmonary vessels (yellow arrows) among the three groups. (J) ∗ versus other groups with different symbols (∗, †, ‡), *P* < 0.001. The scale bars in right lower corners represent 100 *μ*m. HPF = high-power field. Statistical analysis in (D), (E), (F), and (J) using one-way ANOVA, followed by Bonferroni multiple comparison post hoc test. Symbols (∗, †, ‡) indicate significance (at 0.05 level).

H & E staining (Figures 2(A)–2(C)) demonstrated that the number of alveolar sacs was significantly lower in group 2 than that in groups 1 and 3 and significantly lower in group 3 than in group 1 (Figure 2(D)). Conversely, the crowded score showed a significantly opposite pattern compared with that of the number of alveolar sacs among the three groups (Figure 2(E)). Similarly, the number of muscularized pulmonary arterioles showed an identical pattern comparable to that of the crowded score among all groups (Figure 2(F)). Moreover, immunohistochemical staining demonstrated that the differences in the number of small arterioles in lung parenchyma displayed a pattern similar to that of the number of alveolar sacs among the three groups (Figures 2(G)–2(J)).

### 3.4. The Results of Immunofluorescent (IF) Staining of Lung Parenchyma and Pulmonary Artery on Day 28 after Hypoxia-Induced PAH

**Figure 3:**
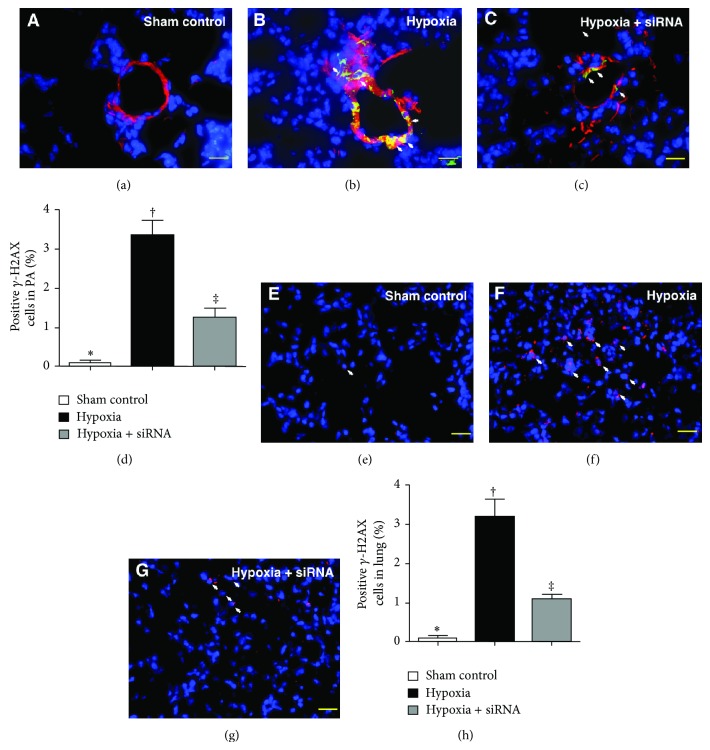
Immunofluorescent (IF) staining for *γ*-H2AX+ cells in medial layer of pulmonary arterioles (PA) and lung parenchyma by day 28 after hypoxia-induced pulmonary arterial hypertension (PAH) (*n* = 10). ((a) to (c)) Identification of *γ*-H2AX+ cells in PA (white arrow) using IF double staining (i.e., *α*-SMA-*γ*-H2AX) in the three groups (400x). (d) ∗ versus other groups with different symbols (∗, †, ‡), *P* < 0.0001. ((e) to (g)) Identification of *γ*-H2AX+ cells in lung parenchyma (white arrow) with IF double staining (i.e., *α*-SMA-*γ*-H2AX) in the three groups (400x). (h) ∗ versus other groups with different symbols (∗, †, ‡), *P* < 0.0001. The scale bars in right lower corners represent 20 *μ*m. Blue fluorescence indicates DAPI-stained nuclei. Statistical analysis in (d) and (h) using one-way ANOVA, followed by Bonferroni multiple comparison post hoc test. Symbols (∗, †, ‡) indicate significance (at 0.05 level).

**Figure 4:**
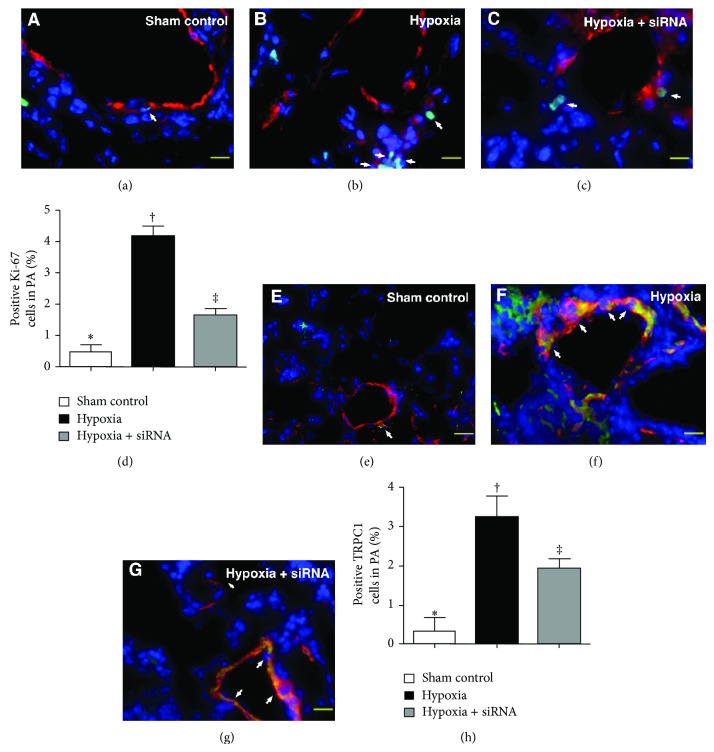
Immunofluorescent (IF) staining for Ki-67+ cells and TRPC1+ cells in medial layer of large pulmonary arterioles (PA) by day 28 after pulmonary arterial hypertension (PAH) (*n* = 10). ((a) to (c)) Identification of Ki-67+ cells in PA (white arrow) with IF double staining (i.e., *α*-SMA-Ki-67) in three groups of animals (400x). (d) ∗ versus other groups with different symbols (∗, †, ‡), *P* < 0.0001. ((e) to (g)) Identification of TRPC1+ cells in PA (white arrow) using IF double staining (i.e., *α*-SMA-*γ*-TRPC1) in the three groups (400x). (h) ∗ versus other groups with different symbols (∗, †, ‡), *P* < 0.0001. The scale bars in right lower corner represent 20 *μ*m. Blue fluorescence indicates DAPI-stained nuclei. Statistical analysis in (d) and (h) using one-way ANOVA, followed by Bonferroni multiple comparison post hoc test. Symbols (∗, †, ‡) indicate significance (at 0.05 level).

IF staining revealed that the number of *γ*-H2AX+ cells in medial layer of large PA and in lung parenchyma (i.e., extra-PA), an index of DNA damage, was significantly higher in group 2 than that in groups 1 and 3 and higher in group 3 than that in group 1 (Figure 3). Besides, IF staining of Ki-67+ cells (i.e., double staining of K-i67 and *α*-smooth muscle actin) (Figures 4(a)–4(d)) for quantifying SMC proliferation in the medial layer of large PA showed a pattern similar to that of *γ*-H2AX+ cells among the three groups. Furthermore, IF staining for identifying TRPC1 (i.e., double staining of TRPC1 and *α*-smooth muscle actin) (Figures 4(e)–4(h)) in the medial layer of large PA showed a prevalence of TRPC1+ cells, an index of hypoxia-inducible biomarker, similar to that of *γ*-H2AX+ cells among the three groups.

**Figure 5:**
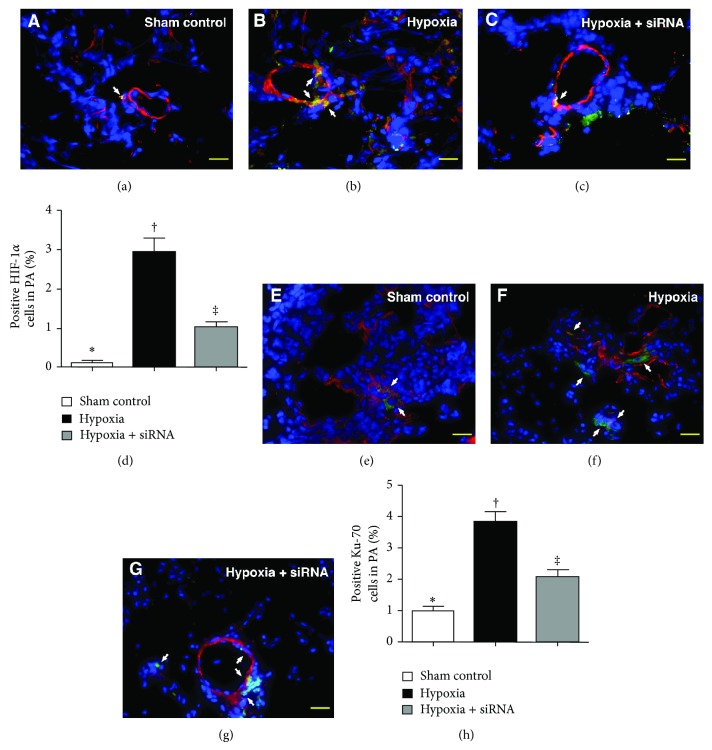
Immunofluorescent (IF) staining for HIF-1*α*+ cells and Ku-70+ cells in medial layer of large pulmonary arterioles (PA) by day 28 after pulmonary arterial hypertension (PAH) (*n* = 10). ((a) to (c)) Cellular expression of hypoxia-inducible factor- (HIF-) 1*α* in PA (white arrow) after double staining (i.e., *α*-SMA-HIF-1*α*) in the three groups of animals (400x). (d) ∗ versus other groups with different symbols (∗, †, ‡), *P* < 0.0001. ((e) to (g)) Identification of Ku-70+ cells in PA (white arrow) using double staining (i.e., *α*-SMA-Ku-70) in the three groups (400x). (h) ∗ versus other groups with different symbols (∗, †, ‡), *P* < 0.001. The scale bars in right lower corners represent 20 *μ*m. Blue fluorescence represents DAPI-stained nuclei. Statistical analysis in (d) and (h) using one-way ANOVA, followed by Bonferroni multiple comparison post hoc test. Symbols (∗, †, ‡) indicate significance (at 0.05 level).

**Figure 6:**
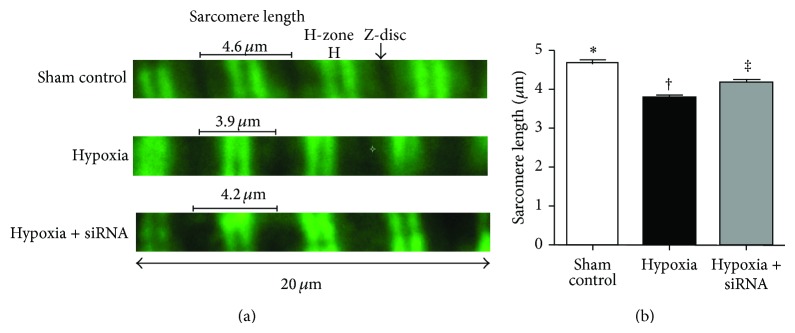
Changes in length of sarcomere in right ventricular myocytes after immunofluorescent (IF) staining by day 28 after pulmonary arterial hypertension (PAH) (*n* = 10). (a) Changes in length of sarcomere identified by troponin-I staining in the three groups (1000x). (b) ∗ versus other groups with different symbols (∗, †, ‡), *P* < 0.001. Statistical analysis using one-way ANOVA, followed by Bonferroni multiple comparison post hoc test. Symbols (∗, †, ‡) indicate significance (at 0.05 level).

Moreover, the same semiquantitative method showed that the prevalence of pulmonary HIF-1*α*+ cells (Figures 5(a)–5(d)), other hypoxia-inducible biomarkers, and Ku-70+ cells (Figures 5(e)–5(h)), an index of DNA repair in nonhomologous end joining pathway in the medial layer of large PA, was similar to that of TRPC1+ cells among the three groups. Furthermore, IF staining of RV cardiomyocytes demonstrated that the length of sarcomere was significantly decreased in group 2 compared to that in groups 1 and 3 and significantly decreased in group 3 than in group 1 (Figure 6). These findings implicated that the hypertrophic RV cardiomyocytes in group 2 exhibited a more rigorous contraction and, therefore, showed a shortened distance between sarcomeres compared to that in the other groups.

### 3.5. Western Blot Analysis of Lung Tissue on Day 28 after Hypoxia Treatment

**Figure 7:**
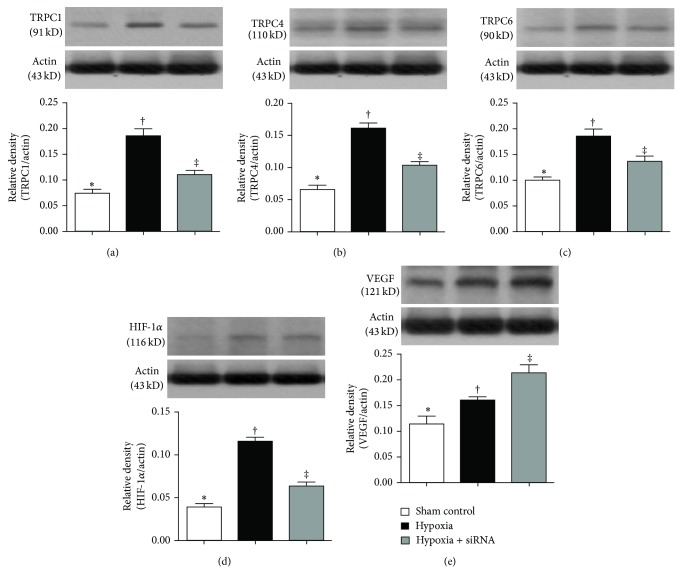
Protein expressions of TRPCs, HIF-1*α*, and VEGF of lung parenchyma by day 28 after hypoxia-induced pulmonary arterial hypertension (PAH) (*n* = 10). (a) Protein expression of TRPC1, ∗ versus other groups with different symbols (∗, †, ‡), *P* < 0.001. (b) Protein expression of TRPC4, ∗ versus other groups with different symbols (∗, †, ‡), *P* < 0.01. (c) Protein expression of TRPC6, ∗ versus other groups with different symbols (∗, †, ‡), *P* < 0.01. (d) Protein expression of HIF-1*α*, ∗ versus other groups with different symbols (∗, †, ‡), *P* < 0.001. (e) Protein expression of vascular endothelial growth factor (VEGF), ∗ versus other groups with different symbols (∗, †, ‡), *P* < 0.01. Statistical analysis in ((a) to (e)) using one-way ANOVA, followed by Bonferroni multiple comparison post hoc test. Symbols (∗, †, ‡) indicate significance (at 0.05 level).

The protein expressions of TRPC1, TRPC4, TRPC6, and HIF-1*α*, four sensitive hypoxia-inducible biomarkers, were significantly upregulated in group 3 and further upregulated in group 2 compared with that in group 1 (Figures 7(a)–7(d)). On the other hand, the protein expression of VEGF, a factor essential for vascular endothelial cell differentiation, was significantly higher in group 3 than that in groups 1 and 2 and significantly higher in group 2 than that in group 1 (Figure 7(e)). This finding implies that depletion of TRPC1 protein expression by siRNA was specific.

**Figure 8:**
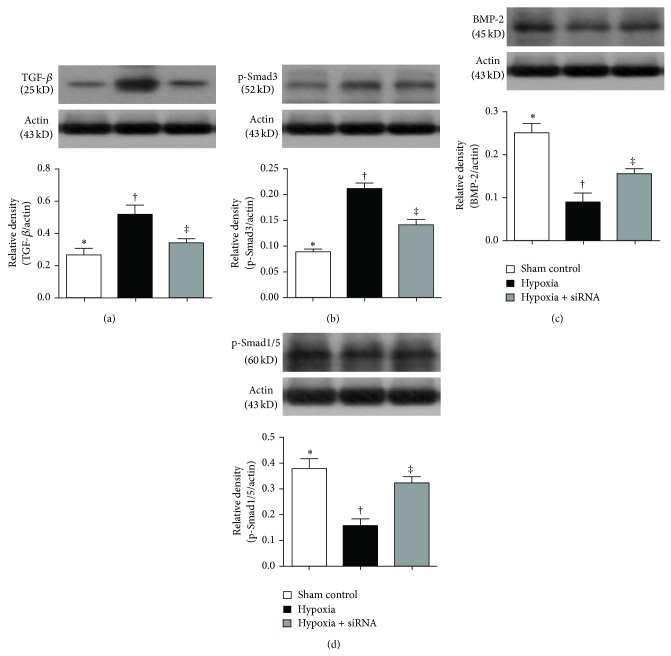
Protein expressions of fibrotic and antifibrotic biomarkers of lung parenchyma by day 28 after hypoxia-induced pulmonary arterial hypertension (PAH) (*n* = 10). (a) Protein expression of transforming growth factor (TGF-*β*), ∗ versus other groups with different symbols (∗, †, ‡), *P* < 0.01. (b) Protein expression of phosphorylated- (P-) Smad3, ∗ versus other groups with different symbols (∗, †, ‡), *P* < 0.001. (c) Protein expression of bone morphogenetic protein- (BMP-) 2, ∗ versus other groups with different symbols (∗, †, ‡), *P* < 0.001. (d) Protein expression of phosphorylated- (P-) Smad1/5, ∗ versus other groups with different symbols (∗, †, ‡), *P* < 0.01. Statistical analysis in ((a) to (d)) using one-way ANOVA, followed by Bonferroni multiple comparison post hoc test. Symbols (∗, †, ‡) indicate significance (at 0.05 level).

The protein expressions of TGF-*β* and Smad3, two indices of fibrosis, were significantly higher in group 2 than that in groups 1 and 3 and significantly higher in group 3 than that in group 1 (Figures 8(a) and 8(b)). By contrast, the protein expressions of BMP-2 and Smad1/5 (Figures 8(c) and 8(d)), two antifibrotic biomarkers, showed an opposite pattern compared to that of TGF-*β* and Smad3 among the three groups.

**Figure 9:**
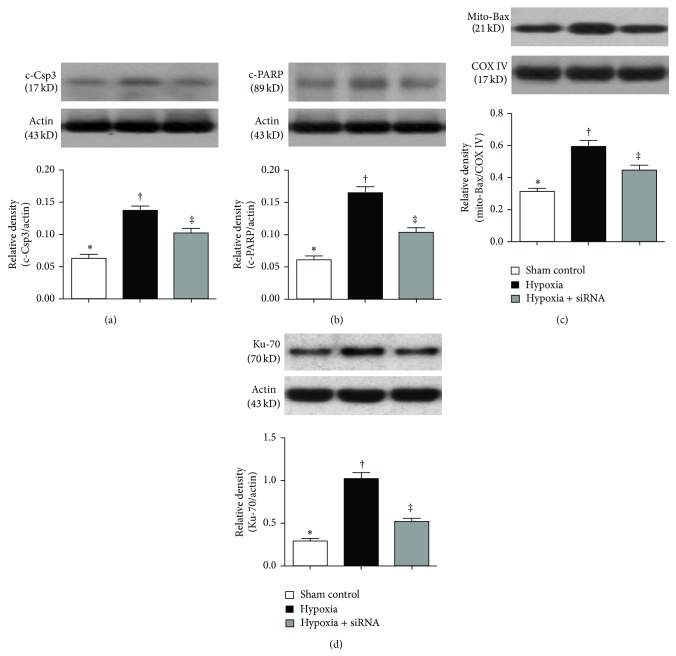
Protein expressions of apoptotic and DNA repair biomarkers in lung parenchyma by day 28 after hypoxia-induced pulmonary arterial hypertension (PAH) (*n* = 10). (a) Protein expressions of cleaved caspase-3 (c-Csp3), ∗ versus other groups with different symbols (∗, †, ‡), *P* < 0.01. (b) Protein expression of cleaved poly(ADP-ribose) polymerase (c-PARP), ∗ versus other groups with different symbols (∗, †, ‡), *P* < 0.001. (c) Protein expression of mitochondrial Bax (mito-Bax), ∗ versus other groups with different symbols (∗, †, ‡), *P* < 0.01. (d) Protein expression of Ku-70, ∗ versus other groups with different symbols (∗, †, ‡), *P* < 0.001. Statistical analysis in ((a) to (d)) using one-way ANOVA, followed by Bonferroni multiple comparison post hoc test. Symbols (∗, †, ‡) indicate significance (at 0.05 level).

The protein expressions of cleaved (i.e., active form) caspase-3 and PARP, two indicators of apoptosis, were significantly increased in group 2 than those in groups 1 and 3 and significantly higher in group 3 than those in group 1 (Figures 9(a) and 9(b)). In addition, the protein expression of mitochondrial Bax, another indicator of apoptotic biomarker, showed a pattern similar to that of cleaved caspase-3 and PARP among the three groups (Figure 9(c)). Furthermore, the protein expression of Ku-70 in lung parenchyma was consistent with the findings from IF staining of these cellular biomarkers in all groups (Figure 9(d)).

### 3.6. Change in Right Ventricular and Pulmonary Gene Expressions on Day 28 after Hypoxia-Induced PAH

**Figure 10:**
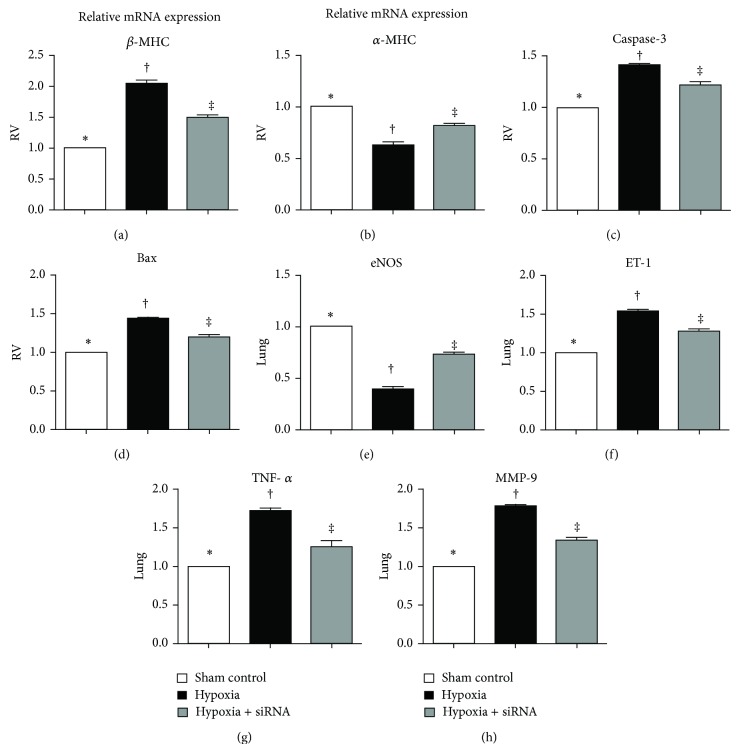
mRNA expressions of fibrotic and apoptotic biomarkers in lung parenchyma and right ventricle (RV) by day 28 after hypoxia-induced pulmonary arterial hypertension (PAH) (*n* = 10). ((a) to (d)) mRNA expressions of (a) *β*-myosin heavy chain (MHC), (b) *α*-MHC, (c) caspase-3, and (d) Bax in RV myocardium. ∗ versus other groups with different symbols (∗, †, ‡), all *P* values in ((a) to (d)) <0.001. ((e) to (h)) mRNA expressions of (e) endothelial nitric oxide synthase (eNOS), (f) endothelin (ET-1), (g) tumor necrotic factor (TNF)-1*α*, and (h) matrix metalloproteinase- (MMP-) 9 in lung tissues. ∗ versus other groups with different symbols (∗, †, ‡), all *P* values in ((e) to (h)) <0.001. Statistical analysis in ((a) to (h)) using one-way ANOVA, followed by Bonferroni multiple comparison post hoc test. Symbols (∗, †, ‡) indicate significance (at 0.05 level).

Cardiac hypertrophy is recognized as a switch from *α*- to *β*-myosin heavy chain (MHC) mRNA expression (i.e., reactivation of fetal gene program). The results of the current study showed that the mRNA expression of *β*-MHC in RV myocardium was notably higher in group 2 than that in groups 1 and 3 and significantly higher in group 3 than that in group 1 (Figure 10(a)). On the other hand, *α*-MHC in RV myocardium was expressed in a significantly reversed manner compared to that of *β*-MHC among the three groups (Figure 10(b)). The mRNA expressions of caspase-3 (Figure 10(c)) and Bax (Figure 10(d)) in RV myocardium, two indicators of apoptosis, showed a pattern similar to that of *β*-MHC in all groups.

Pulmonary mRNA expression of endothelial nitric oxide synthase (eNOS), an index of endothelial functional preservation, was remarkably higher in group 1 than that in groups 1 and 3 and significantly higher in group 3 than that in group 1 (Figure 10(e)). Consistently, the mRNA expression of endothelin- (ET-) 1, an index of endothelial cell dysfunction, showed a significantly opposite pattern compared to that of eNOS (Figure 10(f)). Furthermore, pulmonary mRNA expressions of TNF-*α* (Figure 10(g)) and MMP-9 (Figure 10(h)), two inflammatory biomarkers, were significantly higher in group 2 than those in groups 1 and 3 and significantly higher in group 3 than those in group 1.

## 4. Discussion

This study, which investigated the efficacy of Lipofectamine delivery of siRNA-TRPC1 in suppressing hypoxia-induced PAH, yielded several striking implications. First, Lipofectamine delivery of pEGFP plasmid DNA into the PASMCs was successful. Second, RVSP and TRPC1 expression in PASMCs were remarkably increased by day 28 after hypoxia treatment. Third, successful delivery of siRNA-TRPC1 into the lung through intratracheal spray remarkably reduced the expressions of TRPC1 in PASMCs and lung tissue as well as RVSP.

Although virus-mediated genetic transfer has been utilized for experimental and clinical research [22–24], the safety and effectiveness are still questionable [25, 26]. To develop a delivery system without short-term and long-term side effect, therefore, is of utmost importance in preclinical translational research and also its subsequent clinical application. Studies have recently shown that the Lipofectamine transfection system is superior to the virus-mediated technique for the purpose of safety to the subjects [27, 30]. One important finding in the present study is that Lipofectamine was found to successfully deliver pSuper EGFP into the culturing aortic SMCs* in vitro* and lung tissue* in vivo*. Of importance is that Lipofectamine delivery of siRNA-TPRC1 oligonucleotide into the culturing aortic SMCs successfully suppressed the protein expression of TPRC1.

Previous studies have shown that hypoxia induced the proliferation of PASMCs which plays a principal role in the pathogenesis of PAH [21]. In addition, subfamilies of TRPCs (i.e., TRPC1 to TRPC6) have been demonstrated to be upregulated in mice exposed to hypoxia [21, 38]. One principal finding in the present study is that the expressions of TRPCs (including TRPC1, TRPC4, and TRPC6) and HIF-1*α* were substantially enhanced in hypoxia-treated animals compared to those in the normal controls. Therefore, our findings reinforce those of the previous studies [21, 38]. The most important finding in the present study is that RVSP was remarkably higher in hypoxia-treated animals compared with that in the normal controls and was significantly reduced in hypoxia-treated animals with siRNA-TRPC1 treatment. One particularly important finding is that both upregulations of TRPC1 and HIF-1*α* in hypoxia-treated animals were markedly suppressed in hypoxia animals after siRNA-TRPC1 treatment. Additionally, the number of muscularized PA was found to be notably decreased in hypoxia-treated mice with siRNA-downregulated TRPC1 compared to the hypoxia-treated animals without treatment. Accordingly, our findings may highlight the therapeutic potential of siRNA-TRPC1 in the treatment of PAH patients with poor response to the conventional pharmacological regimen.

Besides, the results of the present study identified that not only the protein expression of TRPC1 but also the protein expressions of TRPC4 and TRPC6 in lung parenchyma were remarkably suppressed by siRNA-TRPC1 treatment. The reason for suppression of the protein expressions of TRPC4 and TRPC6 after siRNA treatment in this experimental setting remains unclear. We propose that the results may be secondary to reduction of PA blood pressure and improvement of hypoxia after treatment which, in turn, led to an inhibition of PASMC proliferation and ultimately a suppression of TRPC4 and TRPC6 expressions.

Another important finding in the present study is that histopathological analysis demonstrated significant reduction in the number of alveolar sacs and notably elevated crowded score in the lung parenchyma of hypoxia-treated animals compared to those in the sham controls. These phenomena, however, were remarkably suppressed in hypoxia-treated animals after siRNA-TRPC1 treatment. In addition, pulmonary fibrotic (TGF-*β*, Smad3) and apoptotic (cleaved caspase-3 and PARP, Bax) biomarkers were also markedly increased in animals after chronic hypoxia but were substantially reduced in the hypoxia-treated animals with siRNA treatment. Furthermore, the number of Ku70+ cells in the medial layer of PA, an indicator of smooth muscle proliferation, was remarkably reduced in hypoxia-treated animals with siRNA treatment compared to those without. Besides, the expression of antifibrotic (BMP-2, Smad1/5) biomarkers in pulmonary parenchyma was significantly higher in hypoxia-treated animals with siRNA treatment than in those without. Using a monocrotaline-induced PAH rat model, our previous studies have demonstrated consistent histopathological findings and expressions of fibrotic and apoptotic biomarkers in pulmonary parenchyma. Accordingly, the findings were significantly improved after treatment with sildenafil [34] or bone marrow-derived endothelial progenitor cells [33]. Therefore, our findings were comparable to those of the previous studies [33, 34]. Importantly, these could, at least in part, explain the attenuation of hypoxia-induced PAH after siRNA-TRPC1 treatment in a murine model.

Previous studies have revealed that VEGF is an important maintaining and differentiating factor for vascular endothelial cells [39] and is also an essential factor for suppressing the development of PAH in experimental models [39, 40]. An interesting finding in the current study is that the protein expression of VEGF was markedly enhanced in hypoxia-treated animals and further augmented after siRNA treatment compared to that in the normal controls. We suggest that there are at least two explanations for the finding. First, the upregulation of VEGF in hypoxia-treated animals might be the result of stress stimulation (i.e., hypoxia conditioning) that enhanced the generation of this biomarker from endothelial cells and SMCs. This may be similar to the induction of HIF-1*α* expression in endothelial cells and SMCs under hypoxic condition. Second, further enhancement of VEGF expression in siRNA-TRPC1-treated animals could be due to the protection offered by siRNA-TRPC1 against endothelial injury, thereby preserving the integrity of endothelial cells which, in turn, secrete more VEGF for their own protection in the present experimental setting. Taken together, our findings, in addition to being supported by those from previous studies [39, 40], also suggest a role of hypoxia-stimulated VEGF production in protecting pulmonary vascular endothelial cells against PAH. Moreover, our results not only imply that the notably reduced RVSP after siRNA treatment may be partially due to increased VEGF production but also indicate the specificity of siRNA in this setting.

Another essential finding in the current study is that *γ*-H2AX, a DNA damage biomarker, and Ki67, a DNA repair activity after DNA damage, were notably increased in the animals after chronic hypoxia-induced PAH and were significantly suppressed after siRNA-TRPC1 treatment. These findings, in addition to being concordant with the histopathological manifestations, could also partially support the significant reduction in hypoxia-induced PAH in animals with siRNA treatment compared to those without.

Intriguingly, pulmonary mRNA expressions of TNF-*α*, MMP-9, and ET-1 were found to be significantly increased, whereas the mRNA expression of eNOS was reduced in hypoxia-treated animals compared to those in the normal controls. These changes, again, were significantly reversed after siRNA treatment. The upregulations of inflammatory and endothelial dysfunction gene expressions in lung parenchyma have also been identified in rats with monocrotaline-induced PAH and were attenuated after sildenafil or endothelial progenitor cell treatment in previous studies [33, 34, 41]. Our findings, in addition to reinforcing those from previous investigations [33, 34, 41], again illustrate the suppression of pulmonary damage and preservation of cardiac function after siRNA-TRPC1 treatment through distinctive histopathological findings in lung parenchyma and attenuation of hypoxia-related RVSP elevation in a murine model of PAH.

Interestingly, the mRNA expressions of Bax and caspase-3 in RV myocardium were significantly increased, whereas the Bcl-2 was significantly lower in hypoxia-treated animals compared with those in the normal controls and was significantly reversed in hypoxia-treated animals after siRNA treatment. One distinctive finding in the RV myocardium is that the *β*-MHC gene expression was notably higher, whereas the *α*-MHC gene expression was significantly lower in animals exposed to chronic hypoxia. In addition, the distance between two sarcomeres was notably shortened in the hypoxia-treated group than in other groups. Fascinatingly, cardiac hypertrophy is characterized by a switch from *α*-MHC to *β*-MHC mRNA expression (i.e., reactivation of fetal gene program) [42, 43]. In this way, our finding is consistent with that of previous studies [42, 43]. Again, the expressions of these genes (i.e., *α*-MHC and *β*-MHC) and the distance between two sarcomeres were markedly reversed in hypoxia animals after siRNA administration. These findings further support those of previous studies [40, 41] and shed light on the mechanisms underlying the improvement in RVSP in hypoxia-treated mice.

## 5. Study Limitations

**Figure 11:**
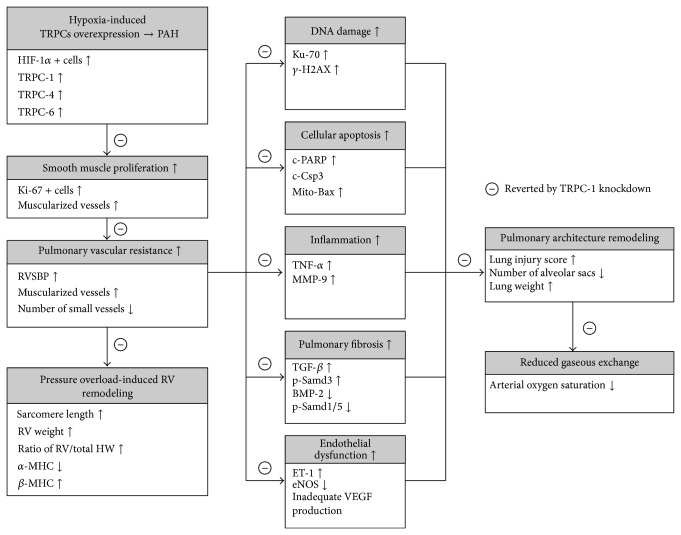
Proposed mechanisms underlying the therapeutic actions of TRPC1 knockdown via Lipofectamine delivered siRNA against hypoxia-induced pulmonary arterial hypertension (PAH) in a murine model of pulmonary arterial hypertension (PAH). *α*-SMA: alpha-smooth muscle actin; BMP-2: bone morphogenetic protein-2; c-Csp3: cleaved caspase-3; c-PARP: cleaved poly(ADP-ribose) polymerase; eNOS: endothelial nitric oxide synthase; ET-1: endothelin-1; HIF-1*α*: hypoxia-inducible factor 1-alpha; HW: heart weight; MHC: myosin heavy chain; mito-Bax: mitochondrial Bax; MMP-9: matrix metalloproteinase-9; p-Smad: phosphorylated Smad; RVSP: right ventricle systolic pressure; TGF-*β*: transforming growth factor beta; TNF-*α*: tumor necrosis factor alpha; TRPC: transient receptor potential cation channel; VEGF: vascular endothelial growth factor.

This study has limitations. First, although the short-term outcome with a study period of only 28 days was promising in the current study, the long-term outcome is still uncertain. Second, this study did not provide information on efficacy and safety of different dosages. Therefore, whether multiple doses would be better than a single dose in the treatment of hypoxia-treatment animals is unknown. Third, due to technical difficulty, this study did not isolate the pulmonary artery for quantification of the protein expressions of TRPC1, TRPC4, and TRPC6. Finally, previously experimental and clinical observational studies have emphasized that the etiologies and the mechanisms of PAH are multifactorial and extremely complicated [1–5, 33, 34]. In the present study, although extensive works had been done, including the molecular-cellular levels of DNA damage, cellular apoptosis, inflammation, pulmonary fibrosis, and endothelial dysfunction, the exact mechanisms of PAH remain uncertain. The proposed mechanisms are summarized in Figure 11 based on our findings.

## 6. Conclusion

The results of this study demonstrated that RVSP and the expressions of TRPCs were significantly enhanced in a murine model of hypoxia-induced PAH and were markedly suppressed after siRNA-TRPC1 treatment. Our findings may provide a therapeutic option with potential clinical applicability in the treatment of patients with PAH refractory to conventional regimens.

## Figures and Tables

**Table 1:** Primers used for real-time PCR amplification.

Gene	GenBank accession number	Forward primer (5′-3′) Reverse primer (5′-3′)	PCR product size (bp)
eNOS	NM_021838-2	TGGAAATTAACGTGGCTGTG GCCTTCTGCTCATTTTCCAA	112

ET-1	M64711.1	TGTCTACTTCTGCCACCTGGA CCTAGTCCATACGGGACGAC	69

TNF-*α*	BC107671.1	GTCTACTGAACTTCGGGGTGA ATGAGAGGGAGCCCATTTG	67

MMP-9	NM_031055.1	AAAAGGCATCCAGCATCTGT AGCTGTCGGCTGTGGTTC	88

*α*-MHC	NM_017239.2	CGAAACTGAAAACGGCAAG TGGCCATGTCCTCGATCT	92

*β*-MHC	NM_017240.1	GCTGCAGAAGAAGCTCAAAGA GCAGCTTCTCCACCTTGG	98

Bax	NM_017059.1	GTGAGCGGCTGCTTGTCT GACTCCAGCCACAAAGATGG	107

Caspase 3	NM_012922.2	AAACCTCCGTGGATTCAAAA AGCCCATTTCAGGGTAATCC	123

Bcl-2	NM_016993.1	GGGATGCCTTTGTGGAACT CTGAGCAGCGTCTTCAGAGA	82

*α*-actin	U19893-1	CTGGGGCCTGAGGAGTTC CCGGTTGAACTCAGCATCA	87

eNOS = endothelial nitric oxide synthase; ET-1 = endothelin-1; TNF-*α* = tumor necrotic factor alpha; MMP = matrix metalloproteinase; MHC = myosin heavy chain; PCR = polymerase chain reaction.

**Table 2:** Baseline characteristics of three group animals at day 28 after hypoxia-induced pulmonary arterial hypertension.

Variable	Group 1 (*n* = 10)	Group 2 (*n* = 10)	Group 3 (*n* = 10)	*P*-value^*^
Body weight (g)	24.91 ± 1.39	25.04 ± 2.32	25.08 ± 1.36	0.977
RBC count (1.0 × 10^6^/mL)	8.14 ± 0.33^a^	10.10 ± 1.05^b^	10.12 ± 1.52^b^	<0.001
Hemoglobulin (gm/dL)	11.83 ± 0.45^a^	14.73 ± 1.52^b^	14.61 ± 2.27^b^	<0.001
RVSP (mm Hg)	20.89 ± 1.69^a^	39.31 ± 3.37^b^	29.57 ± 1.77^c^	<0.001
LVSP (mm Hg)	103.38 ± 10.48	94.96 ± 15.11	94.97 ± 15.07	0.410
RV weight (g)	0.021 ± 0.012^a^	0.028 ± 0.003^b^	0.023 ± 0.025^a^	<0.001
Total HW (g)	0.107 ± 0.404	0.127 ± 0.015	0.119 ± 0.125	0.119
Ratio of RV/total HW	0.178 ± 0.015^a^	0.222 ± 0.302^b^	0.189 ± 0.018^a^	<0.001
Total lung weight (g)	0.145 ± 0.009^a^	0.193 ± 0.027^b^	0.160 ± 0.028^a^	<0.001

Data are expressed as means ± SD.

RBC = red blood cell; RVSP = right ventricular systolic pressure; LVSBP = left ventricular systolic blood pressure; HW = heart weight.

Group 1 = sham control; group 2 = hypoxia + negative control siRNA; group 3 = hypoxia + siRNA-TRPC1.

^*^By one-way ANOVA.

Different letters (a, b, c) are being used for grouping, showing significant difference (at level 0.05) among different groups by Tukey's multiple comparison procedure.
